# Complete genome sequence of *Leucobacte*r sp. WKLB1 isolated from pegmatite in Fukushima, Japan

**DOI:** 10.1128/mra.00219-26

**Published:** 2026-04-20

**Authors:** Momo Sato, Tomoro Warashina, Kazuharu Arakawa

**Affiliations:** 1 Institute for Advanced Biosciences, Keio University, Tsuruoka, Yamagata, Japan; 2 Systems Biology Program, Graduate School of Media and Governance, Keio University 318527, Fujisawa, Kanagawa, Japan; 3 Faculty of Environment and Information Studies, Keio University 120723, Fujisawa, Kanagawa, Japan; University of Southern California, Los Angeles, California, USA

**Keywords:** complete genome, bacteria, nanopore sequencing, *Leucobacter*, pegmatite

## Abstract

We report the complete genome sequence of *Leucobacter* sp. WKLB1, an Actinobacteria isolated from the pegmatite surface in the fourth ore body in the Wagu-Kannon mine, Fukushima Prefecture. The complete circular genome has a genome size of 3.595  Mb, with a G + C content of 66.7%.

## ANNOUNCEMENT

*Leucobacter* is a genus of gram-positive, facultatively aerobic, nonsporulating Actinobacteria with an irregular rod shape, known for its wide distribution across diverse environments (1). On 30 August 2024, we isolated *Leucobacter* strains from the surface of a pegmatite in the fourth ore body of the Wagu-Kannon mine in Fukushima, Japan (37.16400882685028, 140.41975314925645). This mine is known for its large, high-purity mineral crystals. We collected pegmatite samples known to contain several metal-bearing minerals, including schorl, magnetite, chalcopyrite, molybdenite, uraninite, and monazite (2) directly from the mine tunnel using sterilized chisels to prevent contamination. ArsR/SmtB-family transcriptional regulators are known to control responses to elevated levels of various heavy metal ions (3).

To isolate the microbes, we first crushed the pegmatites and suspended them in phosphate-buffered saline (PBS). This suspension was then applied to LB agar medium and incubated at 20°C. We obtained pure colonies by repeatedly picking single colonies from the plates. A single colony was then selected and incubated in LB liquid medium for 64 h. After incubation, the cells were harvested by centrifugation (5,000 × *g*, 5 min, 4°C), and the medium was removed. Genomic DNA was extracted from the lysate using liquid nitrogen and the NucleoBond HMW DNA kit (Macherey-Nagel) by following the manufacturer’s protocol with an extended incubation time of one hour. Long-read sequencing libraries were prepared using the Rapid Barcoding Kit (SQK-RBK114.24, Oxford Nanopore Technologies) without any size selection and sequenced using an R10.4.1 Flowcell (FLO-MIN114) on the MinION Mk1B device using MinKNOW v.24.11.8 (Oxford Nanopore Technologies). 379,541 Sequenced reads (*N*_50_ = 9.223 bp) were basecalled, demultiplexed, and trimmed for adapter sequences using Dorado v.0.9.0 (super accurate mode). Long-read quality was assessed using Nanoplot v1.25.0 (4), and reads longer than 10 kb (approximately 100× coverage) were adapter-trimmed using Porechop v0.2.4 (5) and assembled using Canu version 2.2 (6). The assemblies produced a single chromosome. Overlapping ends were manually deleted to finalize the circularization following the overlapping coordinates specified by the Canu output. Error correction was performed using medaka v.1.11.3 (https://github.com/nanoporetech/medaka) with the adapter-trimmed reads, achieving saturation in completeness in a single round. Assembly completeness was assessed using CheckM v.1.2.2 (7), resulting in a completeness of 99.85% (contamination 1.17%) with o__Actinomycetales (UID1572) as the lineage. The genome was annotated using the DDBJ Fast Annotation and Submission Tool (DFAST) version 1.3.4 (8). The genome was oriented so that the *dnaA* gene was located at the start position by DFAST. Default parameters were used for all software unless otherwise specified. The complete genome of *Leucobacter* sp. WKLB1 has a total length of 3,595,062 bp and contains 3,301 coding genes with GC content percentage of 66.7. Based on the DFAST Taxonomy assignment, the assembled genome of isolate WKLB1 could not be conclusively identified at the species level as *Leucobacter* sp., showing an average nucleotide identity (ANI) of 92.36% to *Leucobacter japonicus* (GCF_001273855.1) and 91.98% to *Leucobacter musarum* subsp. *musarum* (GCF_001273845.1) (9).

The genome of *Leucobacter sp*. WKLB1 contains annotated genes encoding metalloregulator transcription factors, including members of the ArsR and SmtB family, as annotated by DFAST. Investigating the adaptation mechanisms of *Leucobacter* species to the metal-enriched conditions of pegmatites could contribute to understanding their potential role in heavy metal bioremediation within natural ecosystems.

## Data Availability

The genome sequences reported here were deposited in DDBJ under accession number AP046115, and the raw reads were deposited in the Sequence Read Archive (SRA) under BioProject accession number PRJNA1422448 (run as SRR37228286).
